# Systematic review of hydroxychloroquine use in pregnant patients with autoimmune diseases

**DOI:** 10.1186/1546-0096-7-9

**Published:** 2009-05-13

**Authors:** Kirk Sperber, Christine Hom, Chun Peng Chao, Deborah Shapiro, Julia Ash

**Affiliations:** 1 Division of Allergy, Immunology, and Rheumatology, Department of Medicine, New York Medical College, Munger Pavilion, Valhalla, NY 10595, USA; 2 Division of Rheumatology, Department of Pediatrics, New York Medical College, Munger Pavilion, Valhalla, NY 10595, USA

## Abstract

**Objective:**

The purpose of this study is to compare the incidence of congenital defects, spontaneous abortions, number of live births, fetal death and pre-maturity in women with autoimmune diseases taking HCQ during pregnancy.

**Methods:**

The authors searched MEDLINE, Cochrane data base, Ovid-Currents Clinical Medicine, Ovid-Embase:Drugs and Pharmacology, EBSCO, Web of Science, and SCOPUS using the search terms HCQ and/or pregnancy. We attempted to identify all clinical trials from 1980 to 2007 regardless of language or publication status. We also searched Cochrane Central Library and  for clinical trials of HCQ and pregnancy. Data were extracted onto standardized forms and were confirmed.

**Results:**

The odds ratio (OR) of congenital defects in live births of women taking HCQ during pregnancy was 0.66, 95% confidence intervals (CI) 0.25, 1.75. The OR of a live birth for women taking HCQ during pregnancy was 1.05 (95% CI 0.58, 1.93). The OR of spontaneous abortion in women taking HCQ during pregnancy was 0.92 (95% CI 0.49, 1.72). The OR of fetal deaths in women taking HCQ during pregnancy was 0.97 (95% CI 0.14, 6.54). The OR of pre-mature birth defined as birth before 37 weeks in women taking HCQ during pregnancy was 1.10 (95% CI 0.75, 1.61).

**Conclusion:**

HCQ is not associated with any increased risk of congenital defects, spontaneous abortions, fetal death, pre-maturity and decreased numbers of live births in patients with auto-immune diseases.

## Background

The anti-malarial drugs Hydroxychloroquine (HCQ) is widely used to treat various rheumatic diseases [1]. The mechanism of action of this drug is to increase the pH in acidic vesicles, inhibiting receptor mediated endocytosis that affects many cellular functions including antigen presentation, toll receptor signaling and post-transcriptional modification of proteins [2-4]. Convincing evidence has accumulated over the past years demonstrating the efficacy of HCQ in the treatment of auto-immune diseases including systemic lupus erythematosus (SLE), discoid lupus erythematosus (DLE) and rheumatoid arthritis (RA) [5,6]. Since many rheumatic diseases affect women of child bearing age, there have been concerns regarding potential teratogenic and toxic effects of the drug on the developing fetus [7,8].

Malformations and other abnormalities after treatment with higher than the recommended dose of (Chloroquine) CQ through pregnancy were reported after intrauterine exposure to 500 mg daily of CQ in 3 siblings [9]. In animal model systems the drug accumulates in the pigmented cells in the eye [10,11]. Dose-dependent retinal toxicity has long been recognized as the major side effect of HCQ [10,11]. CQ crosses the placenta during pregnancy [12]. In an evidence-based guideline evaluating the risks and benefits of drug therapies during pregnancy, it was recommended to continue HCQ during pregnancy and lactation [13]. Although there have been a number of reviews regarding the use of HCQ during pregnancy [14-16], to date there has been no meta-analysis analyzing the effect of HCQ on fetal outcomes in women with various auto-immune diseases. This systematic review contains a meta-analysis of the available clinical studies investigating the use of HCQ during pregnancy and will focus on the risk of congenital defects, number of live births, spontaneous abortions, fetal deaths and pre-maturity in fetuses born to women taking HCQ.

## Materials and methods

### Study Selection

**Figure 1:**
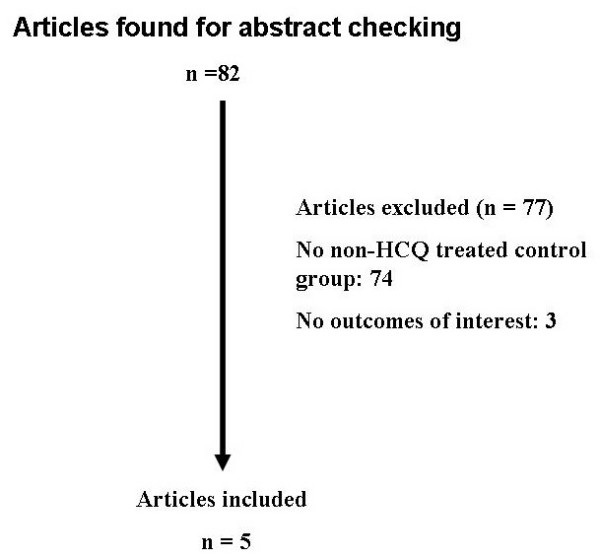
**Identification of relevant articles.** We searched the Cochrane data base, Ovid-Current Contents-Clinical Medicine, Ovid-Embase-Drugs and Pharmacology, EBSCO, Medline, Web of Science, and SCOPUS using the search terms HCQ and/or pregnancy for clinical trials from 1980 to 2007. Potentially relevant studies describing HCQ during pregnancy were retrieved and examined.

We searched the Cochrane data base, Ovid-Current Contents-Clinical Medicine, Ovid-Embase-Drugs and Pharmacology, EBSCO, Medline, Web of Science, and SCOPUS using the search terms HCQ and/or pregnancy. We attempted to identify all clinical trials from 1980 to 2007 regardless of language or publication status. We also searched Cochrane Library and  for clinical trials of HCQ and pregnancy. We also checked the citations of literature reviews and of all trials identified in our search. Potentially relevant studies describing HCQ during pregnancy were retrieved and examined. Figure 1 is a diagram of the process that was used to select the studies included in the meta-analysis. Comparative studies of any design were considered but those studies without a non-HCQ group were excluded. Studies were eligible for inclusion if the investigators had pregnant patients in a non-HCQ treatment group and reported clinical outcomes in the fetus.

### Data Extraction and Quality Assessment

We used previously published methods to determine the quality of the observational studies used in systematic reviews [17,18]. The control groups in the included studies were similar to the HCQ groups for sex, age, criteria for SLE, DLE, RA or other autoimmune diseases as established by American College of Rheumatology (ACR). Two of the authors extracted all of the data onto a standardized form that was confirmed by the corresponding author. The primary outcomes considered in this review were congenital defects, spontaneous abortions, fetal deaths, pre-maturity (birth before 37 weeks of gestation) and live births. Two raters reviewed and scored the articles independently by using a checklist designed by Downs and Black [19]. In cases of divergence of the results between the two raters, a third rater reviewed the articles and rated the quality scores again to choose the most plausible results. The checklist designed by Downs and Black was developed for the purpose of quality assessment of both randomized and nonrandomized studies with health interventions and consists of five subscales: reporting, internal validity bias, internal validity confounding, external validity, and power [19]. Because six items in the original list were related to randomization, and power calculation, their scores were counted as zero. The maximum score on the quality scales is 31 points; these were summed and a higher score is considered to be an indicator of a better quality study. For observational studies, a quality score of 12 or greater is considered excellent [19,20].

### Data Synthesis and Analysis

Data for congenital defects, spontaneous abortions, fetal deaths, pre-maturity (Birth before 37 weeks of gestation) and live births were pooled and analyzed by using the Cochrane Collaboration Review Manager software (Nordic Cochrane Centre, Copenhagen, Denmark) [20]. We used Review Manager 4.2 for the analysis. A fixed effect model of the inverse variance method was used when the effects were assumed to be homogenous. The random effect model of the DerSimonian and Laird approach was used when they were assumed to be heterogeneous [21]. To test the publication bias, funnel plots were drawn with the treatment effect measure on the horizontal axis and the study size on the vertical axis, and Harbord test was performed [22,23]. We used odds ratios (OR) for dichotomous variables in this study. Heterogeneity between studies was assessed by using chi-square and I^2 ^tests, a p value of less than 0.1 and an I^2 ^value of 50% were considered high [24].

We calculated the number of subjects that would have to be included in the meta-analysis for each of the variables, congenital defects, spontaneous abortions, fetal deaths, pre-maturity and live births, to have a power of 0.80 and a one sided p value of 0.05 in pregnant patients taking HCQ [25]. The likelihood that we would be able to detect an association between HCQ and congenital defects, live births, spontaneous abortions, fetal deaths, and pre-maturity is dependent on the magnitude of that association. We can determine an effect size of 0.20 for congenital defects, live births, spontaneous abortions, fetal deaths, and pre-maturity given the number of pregnancies that are included in this analysis (290 pregnancies on HCQ, 341 pregnancy controls) [26].

Data from randomized placebo-controlled trials (RCTs) should not be combined with observational studies in a meta-analysis [17,18]. Since one of the observational studies was a case controlled study, we used odds ratios (OR) for the study variables [24].

### Role of Funding Source

This study was not funded by any outside agency or pharmaceutical company.

## Results

The literature search for HCQ and/or pregnancy identified 82 abstracts. After review of the abstracts and exclusion of non-trials and trials that did not compare pregnant women with auto-immune diseases taking HCQ with a control group, we identified 5 studies of interest [27-31] (Figure 1). The studies were published within the past 15 years. Searches of the Cochrane Library and of the  website did not return any entries of HCQ and pregnancy. This was a safety study which presents considerable difficulties in obtaining sufficient numbers of patients to include in the meta-analysis. Since there are more observational studies than experimental studies in determining the safety of HCQ in pregnancy we included studies of any design for the meta-analysis. [32]. Seventy-seven articles were excluded including 68 with no non-HCQ control group and 3 with no outcomes of interest (Figure 1).

### Quality assessment of the articles

Four of the 5 studies were observational [27-30] and one was a randomized placebo controlled double-blinded trial [31]. Three of the 4 observational studies were published as full manuscripts [27-30] and one was presented as an abstract [30]. One of the observational studies was a case controlled study [30] while the other 3 were cohort studies [27-29]. The first observational study had 69 Lupus patients, 13 unclassified connective tissue disease and 8 patients with primary Sjogren's syndrome [28], the second observational study included 197 women all diagnosed with Lupus [27], the third observational study also followed 86 Lupus patients [29], as well as the fourth observational study 142 women with connective tissue diseases [30] (Additional file 1, Table S1). Only one study was conducted in the United States [27].

The quality of the studies we identified based on the Downs and Black checklist ranged from 20 to 25. Although the double-blinded placebo controlled trail was of the highest quality we did not include it in our analysis [31] for statistical reasons as noted previously [17,18]. We used the Harbord test to investigate funnel plot asymmetry in the analysis because we measured dichotomous variables [23]. This test avoids the mathematical association between log odds ratio and its standard error proposed by Egger et al [18] when there is a substantial intervention effect, while retaining power compared with alternative tests. However, false-positives may occur in the presence of substantial between-study heterogeneity. We did not observe any substantial heterogeneity in the studies in the meta-analysis and no publication bias.

### OR of congenital defects and live births

**Figure 2:**
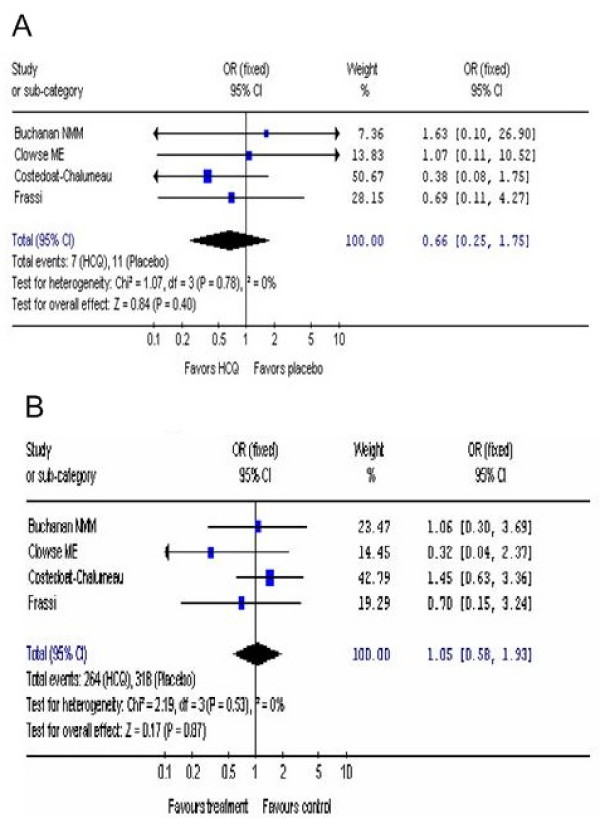
**OR of congenital defects and live births.** The OR for congenital defects (2A)and live births (2B) from the pooled data in the HCQ group compared to the control group was determined.

Other studies have reported that HCQ is safe to use during pregnancy but had no control group [35-40]. All 4 of the studies [27-30] assessed the presence of congenital defects in babies born to women with autoimmune diseases (Additional file 1, Table S2 and Figure 2a). The OR for congenital defects for the pooled data for patients taking HCQ during pregnancy compared to no drug was 0.66 (95% CI, 0.25, 1.75) which favored HCQ (Additional file 1, Table S2 and Figure 2a).

Consonant with the results obtained for the OR of congenital defects, all 4 of the included studies did not report any differences in the OR of live births comparing women who took HCQ compared to the control group (Additional file 1, Table S2 and Figure 2b.) The OR for live births of the pooled data in the HCQ group compared to the control group was 1.10 (95% CI, 0.61, 1.99) (Figure 2b).

### OR of spontaneous abortion and fetal death

**Figure 3:**
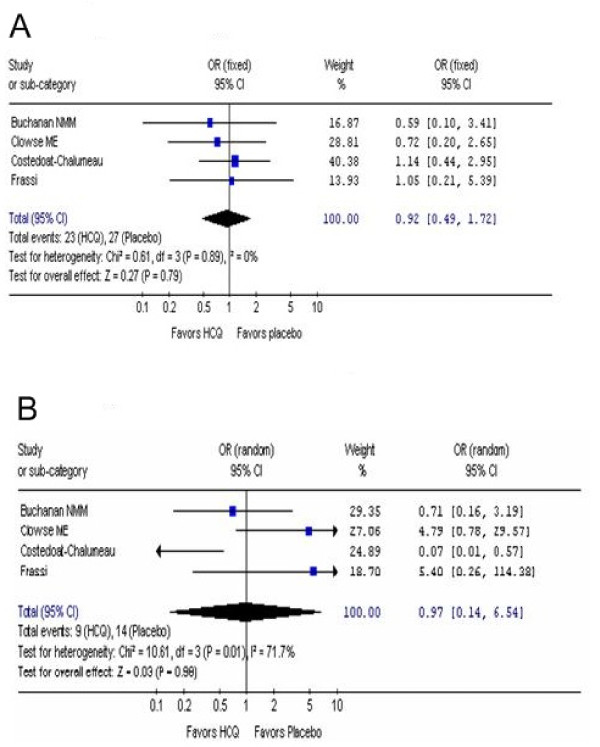
**OR of spontaneous abortion and fetal death.** The pooled estimate of the OR of spontaneous abortion (3A) and fetal death (3B) for the patients taking HCQ compared to placebo was determined.

Women with SLE have a higher incidence of spontaneous abortions and fetal loss [41,42]. Some investigations have shown a correlation between disease activity, spontaneous abortion, and fetal loss in pregnant SLE patients. Different authors have reported a variety of frequencies of spontaneous abortions and fetal loss with rates varying between 11% and 24% [43-47]. Data were available from the 4 studies [27-30] that defined spontaneous abortion and fetal death defined as death occurring after 20 weeks of gestation. The pooled estimate of the OR of spontaneous abortion for the patients taking HCQ compared to placebo was 0.92 (95% CI, 0.49, 1.72) which favored the HCQ group (Additional file 1, Table S2 and Figure 3a).

In line with the results for spontaneous abortions, all 4 studies reported the OR of fetal death [25-28] (Additional file 1, Table S2 and Figure 3b). The pooled OR for fetal death was 0.94 (95% CI 0.14, 6.54) which again favored the patients taking HCQ. There is variability in the fetal death rate probably due to the small sample size.

### OR of prematurity

**Figure 4:**
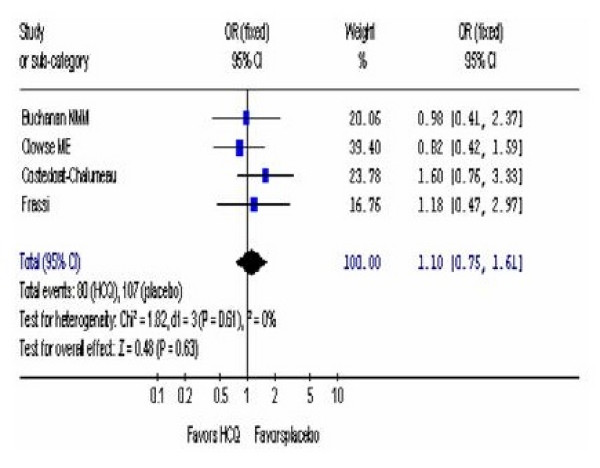
**OR of prematurity.** The pooled estimate of prematurity for patients taking HCQ compared to placebo was determined.

Higher incidences of premature births have been described in SLE patients especially in patients who have active disease [44,45]. Consistent with the results for congenital defects, live births, spontaneous abortions, and fetal deaths, there was no increased risk of pre-maturity associated with HCQ treatment. Data are available from the 4 studies regarding pre-mature death (Additional file 1, Table S2 and Figure 4). The pooled OR was 1.08, (95% CI, 0.74, 1.57).

## Discussion

Our systematic review provides evidence for the use of HCQ during pregnancy for women with auto-immune diseases. We identified 3 cohort studies [27-29] and 1 case-controlled study [30] comparing HCQ treatment with no HCQ treatment in pregnant women with auto-immune diseases (Figure 1 and Additional file 1, Tables S1 and S2). We attempted to identify all relevant studies regarding HCQ and pregnancy by not restricting our literature search to the English language. It is still possible that we may have inadvertently omitted other studies that were not identified in the literature searches of the different databases.

Although the Harbord test for dichotomous variables did not indicate publication bias, citation bias, and database bias, the number of studies included in our meta-analysis was only 4 so that it is possible that it may not be able to distinguish chance from real asymmetry in the funnel plot. The quality of the studies that were included in this meta-analysis as determined by the Downs and Black checklist was excellent despite the fact that they were no randomized placebo-controlled clinical trials [19,20]. The scores are listed in Additional file 1, Table S1.

We did not observe any heterogeneity in the results of the different studies (Figures 2a, b, 3a, and 4) that could have arisen because of differences in doses of HCQ (200 mg compared to 400 mg), length of follow up after birth, and between auto-immune diseases (SLE, DLE, RA, Sjogren's syndrome), allowing the use of a fixed effect model for. The only significant heterogeneous result between the studies was the increased risk of fetal deaths (Figure 3b) noted where the results were analyzed by using a random-effects model.

Our study was limited by the lack placebo controlled double-blinded studies that compared HCQ therapy with a control group. The patient populations included in the studies were fairly homogenous. All of the studies included pregnant females of child bearing age with autoimmune disease predominantly Lupus who met the ACR criteria for diagnosis, all of the patients and the fetuses were exposed to HCQ at the time of conception and throughout the pregnancy, all of the studies had a control group who were not exposed to the drug, and all reported similar outcomes.

We did not include the 1 RCT [31] because of the difficulties of combining different study designs in meta-analysis as noted above [17,18]. This study also found no differences in congenital defects, number of live births, fetal death, and pre-maturity in patients who were treated with HCQ compared to those who were not treated with HCQ. Despite the fact that we performed a meta-analysis, our studies were underpowered to detect an association between congenital defects congenital defects, spontaneous abortions, fetal deaths, pre-maturity and live births, if the effect of HCQ is less than 0.2 [48]. We did consider adding isolated case reports in our analysis given the rarity of the occurrence of congenital malformations and other obstetrical complication of this drug [35-40]. However, we did not include these patients in the statistical analysis because there were no control groups.

There have been numerous studies using CQ prophylaxis for malaria during pregnancy. CQ, a pharmacologic analogue of HCQ has been used in Europe to treat auto-immune diseases and its mechanism of action is identical to HCQ. In general the doses used for malarial prophylaxis are less than those used to treat auto-immune diseases. There is a large meta-analysis published by the Cochrane database that included 12,638 patients focused on malaria prevention [32]. No increase in congenital malformations, live births, spontaneous abortions, fetal deaths or pre-maturity was noted in patients who used CQ compared to controls. Although these studies do not directly prove that CQ is safe for pregnant women with auto-immune diseases, it is further evidence of the safety of anti-malaria drugs during pregnancy.

Two of the included studies by Clowse et. al. [27] and Frassi et al [30] had an increase in fetal death (OR 4.79 95% CI 0.78, 29.57 and OR 5.40 95% CI 0.26,114.28) in the placebo while another study by Costedoat-Chalumeau et al [28] had an OR of 0.08 (95% CI 0.01, 0.60) that favored HCQ. One possible explanation for this discrepancy is the increased number of patients with anti-cardiolipin antibodies in the HCQ group of Clowse et al [27] and Frassi et al [30] that may have contributed to the high incidence of fetal deaths seen in both studies.

Since anti-malarials cross the placenta, they have the potential to cause congenital defects [12]. The first reports documenting congenital abnormalities and accumulation of the drug in the eye were described in infants whose mothers received chloroquine (CQ) [9,33]. However, recent reports including a Cochrane meta-analysis have demonstrated that CQ in low doses is safe as malaria prophylaxis during pregnancy [34]. In addition to the 4 studies that we included in this report, there have been several uncontrolled studies that have examined the effect of HCQ therapy on ocular toxicity in babies born to women taking the drug during pregnancy [35-37]. Two of these studies performed comprehensive ophthalmologic examinations of infants whose mothers took HCQ during pregnancy and found no evidence of ocular toxicity [29,31]. All of these studies have documented the safety of HCQ in infants whose mothers took the medication during pregnancy. The total number of infants examined in these 3 studies is 96.

Another benefit of HCQ during pregnancy is its effect on reducing disease activity. Of note Clowse et al. [27] and Levy et al. [31] made observations of lupus outcomes in a cohort of women with SLE and reported the impact of HCQ treatment cessation on SLE activity during pregnancy. Clowse et al. [27] found that women who stopped taking HCQ had increased lupus activity and increased lupus flares during pregnancy. High activity lupus was defined as a physician's estimate of activity (PEA) greater than 2 occurred in twice as many pregnancies in which HCQ treatment was stopped as in those in which HCQ was continued. The rate of flare was also higher among women who stopped the medication compared with those who either continued taking it or who never took it. These flares occurred throughout pregnancy. More women who discontinued HCQ treatment had SLEDAI scores greater than 4 during pregnancy and this remained elevated when adjusted for year of delivery, anti-phospholipid antibody syndrome (APS), age, ethnicity, and prior history of lupus nephritis.

The types of lupus activity that were best controlled by HCQ were arthritis and constitutional symptoms. HCQ did not prevent the more severe complications of proteinuria or thrombocytopenia. Furthermore, the authors also evaluated the use of Prednisone and fewer women who continued HCQ required high-dose corticosteroids, defined as either a daily dose of Prednisone of at least 20 mg or pulse steroids. Similar to the observation of Clowse et al. [27], Levy et al. [31] noted that after delivery Systemic Lupus Erythematosus Disease Activity Index (SLEDAI) scores of the placebo group were not significantly different from pre-treatment scores while in the HCQ group a statistically significant improvement in the score was found; in the HCQ group there were no flares, and there was a statistically significant improvement in the SLEDAI score at delivery when compared to the placebo group.

## Conclusion

In conclusion, based on the findings of this meta-analysis, HCQ is not associated with any increased risk of congenital defect, spontaneous abortion, fetal death, pre-maturity or decreased numbers of live births in pregnant patients with auto-immune diseases. Our data demonstrate that HCQ is safe for use during pregnancy.

## Competing interests

None of the authors have a financial interest in Sanofi-Winbthrop Pharmaceuticals or any other laboratory that manufactures HCQ.

## Authors' contributions

KS did the literature search, the statistical analysis, and wrote the paper. CH and CPC did the blinded grading and quality assessments of the studies. JA and DS wrote the manuscript.

## Supplementary Material

Additional File 1 **Tables S1 and S2**. Table S1 – Characteristics of the included studies. Table S2 – Data extracted from the included studies and the placebo-controlled trial. Click here for file
