# Supplementary material for: Systematic review of hydroxychloroquine use in pregnant patients with autoimmune diseases
Source: Pediatr Rheumatol Online J. 2009 May 13;7:9. doi: 10.1186/1546-0096-7-9 (PMC2690583; doi:10.1186/1546-0096-7-9)
Supplement: Additional File 1 — Tables S1 and S2. Table S1 – Characteristics of the included studies. Table S2 – Data extracted from the included studies and the placebo-controlled trial. [file 1546-0096-7-9-S1.doc]

**Table I. Characteristics of the Included Studies**

**Total Number**

**of Pregnancies**

**Study, Year Study type on HCQ Study Duration Outcome Measures Results Quality Score**

Buchanan et al (1996) Cohort 36 12 years Congenital defects No increase with HCQ 21

Spontaneous abortion No increase with HCQ

Pre-maturity No increase with HCQ

Live births No increase with HCQ

Fetal deaths No increase with HCQ

Clowse et al. (2006) Cohort 49 15 years Congenital defects No increase with HCQ 24 Spontaneous abortion No increase with HCQ

Pre-maturity No increase with HCQ

Live births No increase with HCQ

Fetal deaths No increase with HCQ

PEA Better disease control with HCQ

SLENDAI Better disease control with HCQ

Costedoat-Chalumeau Cohort 133 9 years Congenital defects No increase with HCQ 25

et al (2003) Spontaneous abortion No increase with HCQ

Pre-maturity No increase with HCQ

Live births No increase with HCQ

Fetal deaths No increase with HCQ

Frassi et al. (2005 ) Case- 76 5 years Congenital defects No increase with HCQ 20

-Controlled Spontaneous abortion No increase with HCQ

Pre-maturity No increase with HCQ

Live births No increase with HCQ

Fetal deaths No increase with HCQ

Studies were excluded from this analysis if they did not contain a control group that did not receive HCQ during pregnancy. The lack of randomization was not an exclusion criterion for the meta-analysis.

HCQ = hydroxychloroquine, PEA = physician estimate of lupus activity, SLENDAI = SLE disease activity index

**Table II. Data Extracted from the Included Studies and the Placebo-Controlled Trial**

**Study, Year Congenital Defects Spontaneous Abortion Pre-maturity Live Births Fetal deaths**

HCQ Placebo HCQ Placebo HCQ Placebo HCQ Placebo HCQ Placebo

Buchanan et al (1996) 1/33 1/53 2/36 4/44 17/36 21/44 28/33 37/44 3/36 5/44

Clowse et al. (2006) 1/49 3/157 3/49 13/157 19/49 64/147 47/49 145/147 3/49 2/149

Costedoat-Chalumeau 3/133 4/70 15/133 7/70 33/133 12/70 117/132 59/70 1/133 7/70

et al (2003)

Frassi et al. (2005 ) 2/76 3/80 3/76 3/77 11/76 10/80 72/76 77/80 2/76 0/80

Levy et al (2001) 0/10 0/10 0/10 1/10 0/10 1/10 10/10 9/10 0/10 1/10

HCQ = hydroxychloroquine
